# Frailty Assessed with FRAIL Scale and G8 Questionnaire Predicts Severe Postoperative Complications in Patients Receiving Major Head and Neck Surgery

**DOI:** 10.3390/jcm11164714

**Published:** 2022-08-12

**Authors:** Viktor Kunz, Gunnar Wichmann, Theresa Wald, Markus Pirlich, Veit Zebralla, Andreas Dietz, Susanne Wiegand

**Affiliations:** Department of Otolaryngology, Head and Neck Surgery, University Hospital Leipzig, 04103 Leipzig, Germany

**Keywords:** head and neck surgery, frailty, G8 questionnaire, FRAIL Scale, postoperative complications, head and neck squamous cell carcinoma, head and neck cancer

## Abstract

Introduction: Frailty represents a complex geriatric syndrome associated with elevated rates of postoperative complications as shown for several malignant entities, including head and neck cancer. A specific screening instrument to assess frailty in head and neck patients does not exist. Both the FRAIL Scale and the G8 questionnaire are well-established and easy to use as screening tools. The present study’s aim was to assess the potential of frailty screening to predict postoperative complications in head and neck patients prior to surgery. Patients and methods: We recorded demographic data, pre-existing medical conditions and clinical characteristics in a prospective cohort of 104 head and neck cancer patients undergoing major head and neck surgery and assessed frailty prospectively on the day of admission utilizing the G8 questionnaire and the FRAIL Scale. We analyzed the link between occurrence of postoperative complications up to the twenty-first postoperative day and age, frailty and other covariates using χ^2^ tests and receiver operating characteristic (ROC) curves. Results: There was no significant correlation between patients’ pre-existing medical conditions and postoperative complications. Whereas chronological age alone did not predict the occurrence of postoperative complications, frailty posed the highest risk for complications. Frailty according to either the G8 questionnaire or the FRAIL Scale predicted occurrence of complications with an area under the curve (AUC) of 0.64 (*p* = 0.018) and 0.62 (*p* = 0.039) and severe complications with an AUC of 0.72 (*p* = 0.014) and 0.69 (*p*=0.031), respectively. Neither frailty score correlated with age or with each other. Conclusion: Prospective screening using the FRAIL Scale or the G8 questionnaire reliably detected frailty in our sample group. Frailty is linked to increased risk of postoperative complications. The correct prediction of severe postoperative complications as shown identifies vulnerable cases and triggers awareness of potential complications. Anticipating risk allows for a more comprehensive view of the patient and triggers decision making towards risk adjustment, and therefore a selective view of alternative treatment modalities.

## 1. Introduction

Population aging and consecutive demographic changes in many societies are challenging healthcare systems worldwide. Frailty is considered the most problematic expression [1] of these trends and is defined as a state of increased vulnerability to acute stressors that can rapidly lead to decompensation under inadequately high stress [1]; for example, stress caused by inadequate medical therapy. Therefore, the presence of frailty in elderly patients not only must be seen as a state of fragility, but also represents a complex geriatric syndrome with high relevance for this group of patients. In this context, correlations between frailty and lower overall survival after critical care [2] and more frequent admission to nursing homes [3] were shown. The presence of frailty as a prognostic factor also plays an important role in cancer patients. Handforth et al. showed significantly elevated rates of complications, severe postoperative complications and mortality in a systematic review of 2916 cancer patients [4]. Therefore, since 2012, the American College of Surgeons National Surgical Quality Improvement Program and American Geriatrics Society guidelines have recommended analyzing the presence of frailty pre-operatively in all elderly patients [5].

Cancer patients, especially patients with head and neck cancer (HNC), represent a distinctly vulnerable group, as these patients tend to be frailer compared to patients with other solid malignancies [6]. In HNC patients who underwent surgery, frailty was shown to be a predictor of severe (Clavien–Dindo Classification ≥ III) complications and higher complication rates independent of age and comorbidity, as well as prolonged hospital stay [7]. The association between frailty in HNC patients and severe postoperative complications was found in previous studies as well [8]. Other factors that emphasize the vulnerability of HNC patients are the association between frailty and functional disorders (swallowing, voice and respiratory disorders) after therapy [9], as well as depression [10]. However, the question is about a robust screening tool that can quickly and easily obtain reliable information about the pre-operative status of neck cancer patients and their potential risk for severe complications associated with frailty.

## 2. Patients and Methods

### 2.1. Statistical Considerations and Sample Size

We prospectively designed a study to evaluate the pretherapeutic frailty screening for detecting frail and pre-frail patients who might be at increased risk for complications. The formula used for sample size calculation along with error margin was as follows:

*n* = (*Z_alpha_*/2)^2^ × *P* × (1 − *P*)/*ε*^2^ (1)

where *Z_alpha_*/2 is the level of significance at 5% = 1.96; *P* = prevalence of pre-frailty and frailty expected (i.e., 40%, or 0.40); and *ε* = desired error of margin (i.e., 15%, or 0.15). So, *n* = (1.96 × 1.96 × 0.40 × (1 − 0.40))/0.15 × 0.15 = 40.98. Thus, expecting a dropout of about 25% caused by either incomplete questionnaires or losses to follow-up for any reason, we chose a sample size of 52 for the study, i.e., 52 pre-frailty or frailty patients as cases and 52 as controls, with 104 patients in total. The study protocol to assess pre-operative frailty and postoperative complications in 104 patients aged ≥65 years was approved by the ethics committee of the Medical Faculty Leipzig (vote 125/22-ek), and the study was executed according to the Declaration of Helsinki.

### 2.2. Study Cohort

**Figure 1:**
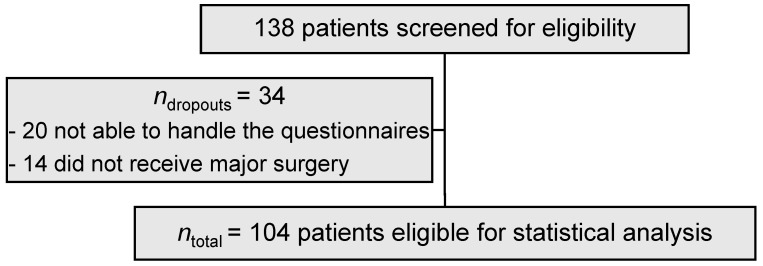
Patient selection.

Pre-operative frailty and postoperative complications were assessed in patients aged ≥65 years who presented to the Department of Otolaryngology, Head and Neck Surgery, University of Leipzig, for major surgery for benign and malign diseases of the head and neck over a period of 9 months. Major surgery, in this context, was defined as a procedure under general anesthesia with the indication for intubation and hospitalization to adequately monitor patients postoperatively. Ambulant procedures were not allowed. Out of 138 pre-screened patients appointed for pre-surgery visits and therefore potentially eligible for frailty screening, 34 patients had to be excluded, so the study cohort was composed of 104 consecutively accrued patients. Out of these 34 excluded patients, 20 were cognitively not able to complete the questionnaires (15 patients had Korsakow’s syndrome, 5 had dementia), and 14 did not receive major surgery. After providing informed consent, the patients were asked to complete the FRAIL Scale and G8 questionnaires on the day of admission as a screening procedure to assess the presence of frailty. All included patients had sufficient knowledge of the German language and could complete the questionnaires on their own. According to the study protocol, we finished the enrollment after recording the 104th patient. Patient selection is shown in Figure 1.

### 2.3. Assessment of Frailty and Postoperative Complications

In our cohort, frailty was assessed using the G8 questionnaire and the FRAIL Scale. The G8 questionnaire consists of 7 queried items, such as nutrition status and amount of medication taken on a daily basis, and can be filled out by patients themselves (self-administered questionnaire). The maximum achievable score is 17 points; a total score of ≤14 defines patients as frail.

The FRAIL Scale consists of five items, including fatigue, illness, ambulation, resistance and loss of weight. Each question corresponds to one item and is scored as 0 or 1 points. Patients are defined as robust only at a score of 0. Pre-frail patients score 1–2 points, and patients are considered as frail at a score ≥ 3 points.

Postoperative complications were observed during a period of 21 days after surgery and classified using the well-established and commonly used Clavien–Dindo classification system. Clavien–Dindo 1 is defined as any deviation from a normal postoperative course, where minor interventions and the use of common drugs, such as analgesics, are allowed. Clavien–Dindo 2 defines complications with the need for drugs or interventions that are not allowed in Clavien–Dindo 1, such as antibiotics or blood transfusions. In this context, Clavien–Dindo 2 was not automatically diagnosed when prophylactic antibiotics were used, but only when wound infections were present. Severe postoperative complications are classified as Clavien–Dindo ≥ 3. In this context, Clavien–Dindo 3 defined complications that needed either surgical or radiological interventions with (a) regional or (b) general anesthesia. Clavien–Dindo 4 stands for life-threatening complications with (a) single or (b) multiple organ dysfunction. The demise of a patient is classified as Clavien–Dindo 5.

### 2.4. Demographic Parameters and Existing Medical Conditions

We assessed and analyzed the influence of demographic parameters, sex, body weight, body mass index (BMI) and chronological age, as well as the occurrence of postoperative complications. The following existing medical conditions were evaluated in our cohort: asthma, chronic obstructive pulmonary disease (COPD), cardiac arrhythmia, hypertension, heart insufficiency, medical history of cardiac infarction, coronary heart disease, diabetes type 1 or 2 and chronic renal insufficiency.

### 2.5. Statistical Analysis

The statistical analysis was performed using the SPSS software package for MacOS (SPSS version 28.0.1, IBM Corporation, Armonk, NY, USA). *Pearson’s* χ^2^ tests, *Mantel–Haenszel* statistics for contingency tables and, where appropriate, *Bonferroni* correction for multiple testing, were used for analysis of categorical covariates. Receiver operator characteristic (ROC) curves were used to investigate the potential of chosen parameters to predict the occurrence of postoperative complications and to find out optimum cut-off values for binary classification. *p* values < 0.05 were considered significant.

## 3. Results

### 3.1. Demographic Parameters and Study Cohort

Our study cohort, eligible for statistical analysis, consisted of 104 patients. A total of 65.4% of these patients were male, and the mean age was 73.4 years. Demographic parameters are summarized in Table 1.

The presence of frailty, existing medical conditions, performed surgical procedures, grouped BMI values and the occurrence of postoperative complications are shown in Table 2.

### 3.2. ROC Analysis

**Figure 2:**
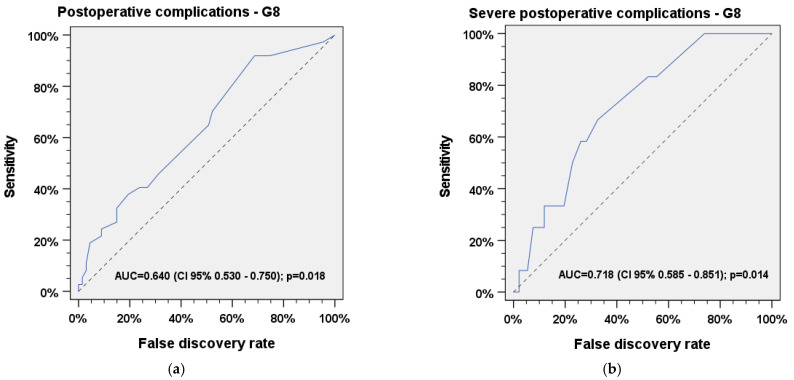
Receiver operating characteristic (ROC) analysis for (**a**) any and (**b**) severe postoperative complications vs. G8 questionnaire.

Frailty according to the G8 questionnaire was found to be a significant predictor of any (AUC = 0.640, 95% CI 0.530–0.750, *p* =0.018) and severe (AUC = 0.718, 95% CI 0.585–0.851, *p* = 0.014) postoperative complications. The optimal cut-off value for all postoperative complications was found at a score of 15 (*Youden’s J* = 0.34). The optimal cut-off value for severe complications was found at a score of 14, which is classified as frailty (*Youden’s J* = 0.45). The ROC curves are shown in Figure 2a,b.

**Figure 3:**
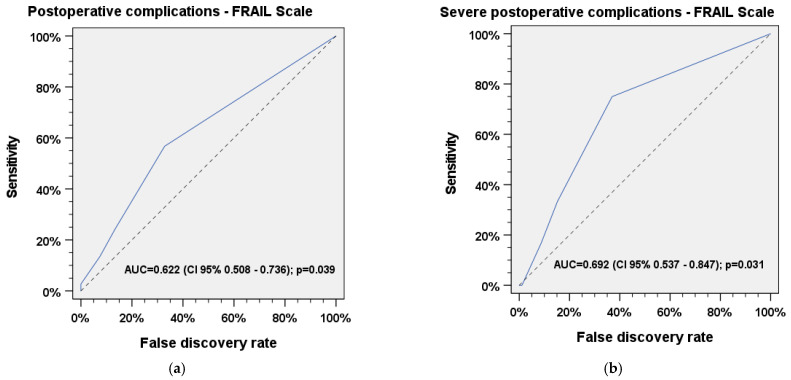
Receiver operater characteristic (ROC) analysis for (**a**) any and (**b**) severe postoperative complications vs. FRAIL Scale.

Frailty according to the FRAIL Scale also predicted occurrence of any (AUC = 0.622, 95% CI 0.508–0.736, *p* = 0.039) and severe (AUC = 0.692, 95% CI 0.537–0.847, *p* = 0.031) postoperative complications. The optimal cut-off values for any and severe complications were found at a score of 1, which indicates pre-frailty (*Youden’s J* = 0.38 and *Youden’s J* = 0.47, respectively). The ROC curves are shown in Figure 3a,b.

None of the medical conditions assessed were found to be significant predictors for occurrence of any or severe postoperative complications in our cohort. Chronological age, body weight and BMI did not have an association with the occurrence of postoperative complications and were not found to be a significant predictor of outcomes among patients in our cohort.

χ^2^ test

According to the results of the ROC analysis, we decided to summarize pre-frail and frail patients (according to the FRAIL Scale) into one group, as our data above showthat even pre-frail individuals were significantly more likely to develop postoperative complications.

Our results show that the occurrence of any (χ^2^ = 7.11, *p* = 0.008, φ = 0.26) and severe postoperative (χ^2^ = 19.51, *p* ≤ 0.001, φ = 0.43) complications was significantly more likely in patients with HNSCC.

We found a significant coherence between the occurrence of frailty according to the G8 questionnaire and the FRAIL Scale in our cohort (χ^2^ = 4.46, *p* = 0.035, φ = 0.25).

There was a significant correlation between postoperative complications in general and the presence of frailty, according to the FRAIL Scale for all patients (χ^2^ = 4.46, *p* = 0.035, φ = 0.21, PPV = 47.62%, NPV = 72.58%), as well as HNSCC patients (χ^2^ = 3.95, *p* = 0.047, φ = 0.33, PPV = 68.42%, NPV = 64.71%). We found no significant association between the occurrence of postoperative complications and the presence of frailty, according to the G8 questionnaire (*p* = 0.26). There also was no significant correlation between mild postoperative complications and frailty according to the G8 questionnaire (*p* = 0.832) and the FRAIL Scale (*p* = 0.673). A significant correlation was found between severe postoperative complications and the presence of frailty according to the FRAIL Scale (χ^2^ = 6.75, *p* = 0.009, φ = 0.26, PPV = 21.43%, NPV = 95.16%) and the G8 questionnaire (χ^2^ = 4.75, *p* = 0.029, φ = 0.21, PPV = 17.86%, NPV = 95.83%).A significant correlation was found between the presence of frailty according to the G8 questionnaire and hypertonia (χ^2^ = 9.32, *p* = 0.002, φ = 0.3), as well as diabetes (χ^2^ = 8.32, *p* = 0.004, φ = 0.28), and between COPD and frailty, according to the FRAIL Scale (χ^2^ = 7.21, *p* = 0.007, φ = 0.26). A significant correlation could also be demonstrated between the presence of cardiac arrhythmia and HNSCC (χ^2^ = 5.42, *p* = 0.020, φ = 0.22). No significant coherence was found between all existing medical conditions and the occurrence of postoperative complications (any, mild, severe), as well as all demographic parameters assessed. The results of the χ^2^ tests are summarized in Table 3, Table 4 and Table 5.

## 4. Discussion

ROC analyses indicate that both the G8 and the FRAIL Scale are capable to significantly predict frailty and the frailty-associated risk of postoperative and severe postoperative complications in patients who received major head and neck surgery. Regarding severe postoperative complications, both questionnaires seem to be suitable tools for reliable prediction (AUC_FRAILScale_ = 0.69, *p*= 0.031; AUC_G8_ = 0.72, *p* = 0.014). We found an optimal cut-off value of 15 in the G8 questionnaire for prediction of all postoperative complications, which would be considered as *robust* and should be considered during evaluation of the G8 questionnaire when screening for frailty is performed. An optimal cut-off value of 14 in the G8 questionnaire was found to predict severe postoperative complications. This value was considered as *frail*, indicating that frail patients were more likely develop severe postoperative complications after elective major head and neck surgery. ROC analysis of the FRAIL Scale shows that even pre-frail patients with a total score of 1 have a significantly increased risk for postoperative and severe postoperative complications. In this context, classifying individuals as pre-frail rather than frail does not seem to have any benefit, considering the fact that both groups are likely to develop severe postoperative complications. Therefore, the inclusion of the FRAIL Scale results prior to surgery in the decision-making process for head and neck patients is strongly recommended.

Regarding general postoperative complications, the χ^2^ test shows a significant correlation with the presence of frailty according to the FRAIL Scale. This screening instrument seems to be a good tool for estimating the occurrence of all postoperative complications, even when pre-frailty is present in head and neck patients.

The χ^2^ tests show that the occurrence of any and severe postoperative complications is significantly more likely in HNSCC patients compared to other ENT diagnoses. As we were able to show that this subgroup does not have significantly more existing medical conditions than patients with other ENT diagnoses, a possible explanation for these results may be the more invasive and prolonged surgery for treatment of HNSCC.

The χ^2^ tests reveal a significant coherence between the presence of frailty according to both questionnaires and severe postoperative complications. Considering the results of ROC analysis for prediction of severe postoperative complications, both questionnaires are good screening instruments for detecting frailty in head and neck patients prior to elective major surgery. Their outcomes can trigger awareness for frailty-associated risk and anticipate the increased occurrence of severe postoperative complications whenever frailty is present. The high NPVs of both questionnaires also implicate a high probability for an uncomplicated postoperative course when frailty is not present in assessment before major head and neck surgery. To confirm these findings, a prospective validation study with a larger cohort of particular subgroups of ENT patients is needed in the future, as our cohort may not be representative due to selection bias or imbalanced gender distribution.

Chronological age does not predict any, mild or severe postoperative complications, and it is also not significantly linked to the occurrence of any postoperative complications. This confirms the results of earlier studies [7,11] and a recent meta-analysis [12] implicating that the complexity of frailty cannot be explained or depicted by chronological age alone, and extends this knowledge to any major surgery in the head and neck region.

Previous studies found a significant correlation between frailty and cardiac diseases [13], as well as frailty and pulmonary diseases [14]. Among our patients, none of the assessed pre-existing medical conditions alone was significantly associated with any, mild or severe postoperative complications, meaning that in our cohort, the presence of frailty marked the only relevant risk factor for complications.

Interestingly, we did not find a significant correlation between the G8 questionnaire and the FRAIL Scale regarding the presence of frailty in HNSCC patients. These results show an inconsistency between these two screening tools in detecting the presence of frailty. A plausible explanation for this result might be the relatively small sample size of only 36 HNSCC patients, compared to 68 non-HNSCC patients. In this context, statistically significant coherences in HNSCC patients might have been present but not detectable due to the smaller size of this sub-cohort. Another interpretation of these findings might be that neither of the two questionnaires was specifically developed for HNC patients, and therefore might not fully reflect or assess the specific functional problems and vulnerability of this special group of patients. As indicated by this particular result, there is not an optimal frailty screening tool available for either head and neck patients or for HNC patients. At the same time, the positive predictive value of frailty for postsurgical complications and severe complications in particular highlights that such an instrument is very desirable for this highly vulnerable group of patients. Further research may be needed to develop and evaluate a short and easy-to-use frailty screening for HNC patients in clinical routine. As long as no screening tool exists that can reliably address the specific and so far unmet need for detection among HNC and other ENT patients undergoing major surgery, those patients will be at increased risk for complications. Frailty screening using the G8 questionnaire or the FRAIL Scale is advised.

### Limitations

Our study has some limitations that need to be discussed. First, our study included biases because the characteristics of the unselected consecutively accrued patients, such as sex, reason for surgery and, most importantly, treatment received, were heterogeneous. Patients with the full spectrum of underlying diseases in the head and neck area, who required different surgical procedures, were included to achieve the most comprehensive view on frailty and its relevance for postsurgical outcome, and to provide a broad unselected “real life” data set for all head and neck patients. Naturally, these procedures may vary in their complication rates, which may also be caused by different durations of the procedures. This bias is important to highlight, as it might influence the predictive value of frailty for postoperative complications, especially in very long cancer surgery procedures with the need for reconstruction. Second, this study included a relatively low number of HNSCC patients compared to the patients who were treated for benign diseases, reducing the probability of complications and hence their prevalence. Furthermore, there could have been underreporting of complications, as we are a tertiary medical center and we might have missed lower-grade postoperative complications whenever such patients were readmitted to other hospitals or treated by another healthcare practitioner.

## 5. Conclusions

In our study, frailty was diagnosed correctly to the expected extent. Chronological age and the presence of frailty differed significantly in predicting postoperative complications, showing that pre-operative frailty and pre-frailty predict increased risk for severe postoperative complications, but not chronological age. The G8 questionnaire and the FRAIL Scale are both suitable screening instruments for anticipating the occurrence of postoperative complications.

Frailty screening enables us to identify vulnerable cases and can help us to raise awareness of risk for potentially occurring complications. As anticipating risk allows preparation for adequate and timely counteractions, including the possibility of switching to alternative treatment modalities, we recommend using the FRAIL Scale or the G8 questionnaire to facilitate decision-making before major head and neck surgery.

## Figures and Tables

**Table 1:** Demographic parameters of the study cohort, HNSCC and other ENT diagnoses.

		All Patients	HNSCC	Other	*p*
Age	*n*	104	36	68	
	Minimum	65	65	65	
	Maximum	88	84	88	
	Mean	73.41	72.69	73.79	n.s.
	SD	6.07	5.31	6.44	
Weight	*n*	104	36	68	
	Minimum	47	47	54	
	Maximum	169	104	169	
	Median	77.0	75	78	
	Mean	78.94	75.47	80.78	n.s.
	SD	17.23	15.67	17.84	
Sex	*n*	104	36	68	
	Male (%)	68 (65.4%)	23 (63.9%)	45 (66.2%)	n.s.
	Female (%)	36 (34.6%)	13 (36.1%)	23 (33.8%)	n.s.

HNSCC: head and neck squamous cell carcinoma; ENT: ear, nose, throat; n.s.: not significant.

**Table 2:** Descriptions of patients’ characteristics, surgical procedures and postoperative complications.

Frailty	* n *	%
G8	56	53.8
FRAIL Scale	42	40.4
**Existing medical conditions**		
Asthma	2	1.9
COPD	10	9.6
Cardiac arrhythmia	22	21.2
Hypertension	62	59.6
Heart insufficiency	6	5.8
Myocardial infarction in medical history	2	1.9
Coronary heart disease	8	7.7
Diabetes type 1 and 2	32	30.8
Dementia	1	0.96
**BMI**		
<19	6	5.8
19–21	4	3.8
21–23	6	5.8
≥23	88	84.6
**Diagnoses with indication for major surgery**		
HNSCC (larynx, pharynx, oral cavity, neck)	36	34.6
Benign lesions of the larynx	13	12.5
Benign lesions of the lymphatic nodes (neck)	6	5.8
Skin lesions/skin tumors (benign and malignant)	5	4.8
Subglottic/tracheal stenosis	2	1.9
Benign tumors or lesions of the salivary glands	10	9.6
Septum deviation, chronic sinusitis	12	11.5
Hearing loss, chronic mesotympanal/epitympanal otitis media	11	10.6
Benign lesions of the pharynx/oral cavity, chronic tonsillitis	4	3.8
Esophageal stenosis, Zenker’s diverticulum	5	4.8
**Postoperative complications**		
Any	37	35.6
Mild (Clavien–Dindo ≤ 2)	25	24.0
Severe (Clavien–Dindo ≥ 3)	12	11.5
**Grouped surgical procedures**	*** n ***	** Mean time (min) **
Cancer surgery in HNSCC (pharynx, larynx, oral cavity, neck dissection)	36	298.25
Without reconstruction	25	204.56
With reconstruction (radial forearm flap, anterior lateral thigh flap, pectoralis major flap)	11	511.18
Septoplasty/FESS/pansinus surgery	12	64.92
Mikrolaryngoscopy for benign lesions with laser	13	34.07
Excision of skin lesions (benign and malignant)	5	99.68
Laryngotracheal reconstruction	2	100.06
Partial parotidectomy/submandibulectomy	10	101.0
Cochlear implant/tympanoplasty	11	78.09
Excision of lesion (benign) pharynx/oral cavity, tonsillectomy	4	31.5
Esophageal bougienage (three sessions), endoscopic laser diverticulotomy	5	32.6
Lymphatic node extirpation (neck)	6	42.46

HNSCC: head and neck squamous cell carcinoma; COPD: chronic obstructive pulmonary disease; BMI: body mass index, kg/m^2^.

**Table 3:** χ^2^ test for occurrence of postoperative complications and occurrence of postoperative complications depending on frailty.

Postoperative Complications		χ^2^	*p*	φ
HNSCC vs. other	Any	7.11	0.008	0.26
	Mild	0.099	0.752	0.03
	Severe	19.51	<0.001	0.43
**Any postoperative complications and frailty**				
FRAIL Scale	All patients	4.46	0.035	0.21
	HNSCC	3.95	0.047	0.33
	Other	0.281	0.596	0.06
G8	All patients	1.59	0.206	0.12
	HNSCC	0.892	0.345	0.16
	Other	0.085	0.771	0.04
**Mild postoperative complications and frailty**				
FRAIL Scale	All patients	0.179	0.673	0.04
	HNSCC	0.390	0.532	0.10
	Other	0.022	0.882	0.02
G8	All patients	0.045	0.832	0.02
	HNSCC	0.080	0.778	0.05
	Other	0.000	01.00	0.00
**Severe postoperative complications and frailty**				
FRAIL Scale	All patients	6.75	0.009	0.26
	HNSCC	2.53	0.112	0.27
	Other	1.97	0.159	0.17
G8	All patients	4.75	0.029	0.21
	HNSCC	1.64	0.201	0.21
	Other	1.14	0.285	0.13

HNSCC: head and neck squamous cell carcinoma.

**Table 4:** χ^2^ test for presence of frailty and existing medical conditions.

Frailty	Existing Medical Conditions	χ^2^	*p*	φ
FRAIL Scale	Asthma	0.078	0.780	0.03
	COPD	7.27	0.007	0.26
	Cardiac arrhythmia	0.298	0.585	0.05
	Hypertension	0.153	0.695	0.04
	Heart insufficiency	0.245	0.621	0.05
	Myocardial infarction in medical history	1.38	0.240	0.12
	Coronary heart disease	0.030	0.863	0.02
	Diabetes type 1 and 2	1.76	0.183	0.13
G8	Asthma	0.012	0.912	0.01
	COPD	1.16	0.281	0.11
	Cardiac arrhythmia	0.309	0.578	0.05
	Hypertension	9.32	0.002	0.30
	Heart insufficiency	2.23	0.136	0.15
	Myocardial infarction in medical history	2.38	0.123	0.15
	Coronary heart disease	1.56	0.212	0.12
	Diabetes type 1 and 2	8.32	0.004	0.28

HNSCC: head and neck squamous cell carcinoma; COPD: chronic obstructive pulmonary disease.

**Table 5:** χ^2^ test for frailty and existing medical conditions between HNSCC and other ENT diagnoses.

Frailty (HNSCC vs. Other)	χ ^2^	* p *	φ
G8	3.64	0.056	0.19
FRAIL Scale	3.51	0.061	0.18
**Existing medical conditions (HNSCC vs. other)**			
Asthma	1.08	0.299	0.10
COPD	3.15	0.076	0.17
Cardiac arrhythmia	5.42	0.020	0.22
Hypertension	0.418	0.518	0.06
Heart insufficiency	3.37	0.066	0.18
Myocardial infarction in medical history	0.213	0.644	0.04
Coronary heart disease	1.87	0.171	0.13
Diabetes type 1 and 2	0.860	0.354	0.09

HNSCC: head and neck squamous cell carcinoma; COPD: chronic obstructive pulmonary disease.

## Data Availability

The data sets used and/or analyzed during the current study are available from the corresponding author upon reasonable request.

## Abbreviations

BMI	Body mass index
Clavien–Dindo	Classification system for postoperative complications
COPD	Chronic obstructive pulmonary disease
FRAIL Scale	Screening instrument for the assessment of frailty
G8	Geriatric eight (screening instrument for the assessment of frailty)
HNC	Head and neck cancer
HNSCC	Head and neck squamous cell carcinoma
IBM	International Business Machines Corporation
*n*	Sample size
NPV	Negative predictive value
*p*	*p* value
PPV	Positive predictive value
ROC	Receiver operating characteristic (used to assess the accuracy of tests in predictive models)
SD	Standard deviation
SPSS	Statistical package for the Social Sciences
φ	Cramer’s phi (measure of effect size in χ² test)
